# The Pericardium Cells Junctions Are a Target for Autoantibodies of Patients Affected by a Variant of Endemic Pemphigus Foliaceus in El Bagre and Surrounding Municipalities in Colombia, South America

**DOI:** 10.3390/diagnostics15080964

**Published:** 2025-04-10

**Authors:** Ana Maria Abreu Velez, Takashi Hashimoto, Yulieth A. Upegui, Jorge Mario Vélez Arango, Adriana Milena Olarte Aponte, Jose A. Vega, Michael S. Howard

**Affiliations:** 1 Georgia Dermatopathology Associates, Atlanta, GA 30329, USA; 2 Department of Dermatology, Graduate School of Medicine, Osaka Metropolitan University, Osaka 545-8585, Japan; hashyt@gmail.com; 3 Research Group in Infections and Health in the Tropics, School of Medicine, Universidad Nacional de Colombia, 16486 Bogota, Colombia; yupegui@unal.edu.co; 4 Diagnostic Aids, Research Group on Images & AI in Health, SURA, Cra. 43A#5A-113, Medellín, Colombia; jorgemariovelezarango@gmail.com; 5 Program in Basic Sciences, Universidad Colegio Mayor de Cundinamarca (UCMC), 110311 Bogota, Colombia; amilenaolarte@unicolmayor.edu.co; 6 Departamento de Morfología y Biología Celular, Grupo Sistema Nervioso Periférico y Órganos de los Sentidos, Universidad de Oviedo, 33006 Oviedo, Spain; 7 Facultad de Ciencias de la Salud, Universidad Autónoma de Chile, Providencia Área Metropolitana, 7500912 Santiago, Chile

**Keywords:** endemic pemphigus foliaceus in El Bagre, epicardium, pericardium, sensory nerve formations, material transfer agreement

## Abstract

**Background:** Patients suffering from a new variant of endemic pemphigus foliaceus in El Bagre, Colombia, South America (El Bagre-EPF) produce autoantibodies (Abs) to different proteins in the skin (frustre form), as well as to those in other organs (Senear–Usher-like and systemic forms). Here, we hypothesize whether patients’ autoantibodies play a role in triggering epicardium and pericardium autoimmunity and pathogenicity. We based this hypothesis on knowing that these patients frequently show clinical symptoms of the chest and heart, and we hypothesize that the autoantibodies of this disease are the main contributors to the base of the pericardial conditions of these patients. **Materials and Methods**: A case-control study for testing the sera of patients affected by El Bagre-EPF (n = 45) and matched controls from the endemic area (n = 45) was conducted to evaluate reactivity with the pericardial tissue. Patients’ necropsies were tested by immunohistochemistry (IHC), in El Bagre-EPF patients (n = 7) and matched controls. **Results:** The sera from most El Bagre-EPF patients displayed polyclonal autoreactivity with both layers of the pericardium, i.e., fibrous pericardium and serous pericardium (mainly to cell junctions and sensory nerve formations), as well as with the neurovascular cell junction branches. Controls were negative (*p* < 0.1). These reactivities were detected by IIF, CM, and IHC using secondary Abs against total IgG, IgM, Kappa and lambda, C3C of the complement, fibrinogen, and albumin. Furthermore, Abs against MIZAP, ARVCF, desmoplakin I-II, and p0071 colocalized with the Abs of El Bagre-EPF (*p* < 0.1). **Conclusions**: Patients affected by El Bagre-EPF produce autoantibodies directed against molecules present in the cell junctions of the pericardium and adnexal structures. Further studies will focus on the clinical significance of these findings.

## 1. Introduction

Endemic pemphigus foliaceus (EPF) or South American pemphigus foliaceus (A.K.A. Fogo Selvagem, wildfire) is an orphan autoimmune blistering disease, which is considered to be endemic in some areas in Brazil and central and south American countries [1,2,3,4,5]. The occurrence of EPF has also been reported in Africa, particularly in south Tunisia, which raises some concerns due to its apparent association with pregnancy and/or postpartum [5,6,7,8,9].

We identified a new variant EPF in El Bagre, a region of the State of Antioquia (Colombia, South America) and neighboring geographic areas bordering with other states. We denominated this variant as El Bagre-EPF (A.K.A. Pemphigus Abreu-Manu) and performed extensive research to characterize this disease [10,11,12,13].

It is currently accepted that EPF is an organ-specific skin disease. Nevertheless, accumulating evidence revealed systemic involvement in patients affected by both variants of EPF in Brazil (AKA fogo selvagem) and El Bagre-EPF [2,3,4,5,14,15,16]. In the El Bagre-EPF variant, the autoantibodies recognize various proteins, particularly, cell-junction proteins in the skin in frustre form (localized only in the seborrheic areas of the face and chest) and proteins in non-skin organs in the intermediate form between the frustre form and Senear–Usher-like systemic (resembles a mix of pemphigus and lupus) generalized form, affecting more than 70% of the skin. The systemic generalized form shows erythrodermic, bullous, exfoliative, and hyperkeratotic skin lesions, and also affects various endocrine glands, the cardiovascular system, muscles, kidneys, and other organs [10,11,12,13,16]. Furthermore, clinical evidence suggested that some chest organs are also affected in El Bagre-EPF patients. In fact, many patients show episodes of sudden chest pain, dyspnea, hiccups, dysphagia, palpitations, fatigue, anxiety, confusion, and syncope. Therefore, the present study was designed to investigate whether the pericardium and its neurovascular system are also targets for the autoantibodies in El Bagre-EPF patients and whether the autoantibodies of these patients are at the basis of the etiopathogenesis of the pericardial manifestations of the disease.

## 2. Materials and Methods

### 2.1. Patients

The study was approved by the Ethical Committee of the Hospital Nuestra Señora del Carmen, in El Bagre, Antioquia, Colombia, and it was conducted in agreement with the guidelines of the Declaration of Helsinki II. All participants signed informed consent. We examined 45 patients affected by El Bagre-EPF and 45 controls without known autoimmune diseases from the endemic area matched by age, gender, demographics, comorbidities, and work activities. No identifier was presented in the study, nor the data, from each patient, ensuring ethical research practices and building trust. This involves securing information, limiting access, and obtaining informed consent, with exceptions for legal or mandatory reporting. Investigators secure data storage: data, both electronic and physical, is being maintained securely in locked cabinets and with password-protected systems. Additionally, anonymization using alphanumeric codes instead of full names can protect participant identity. Only researchers have access to participant data and all investigators have and retain confidentiality protocols and data security measures. The study was conducted based on the data obtained from the clinical evaluation since no radiological study was available (see justification in the Discussion section).

### 2.2. Clinical Evaluation

Clinical examinations include the following signs and symptoms: pericardial rub on auscultation, PR depression, ST segment deviation, widespread ST-elevation and PR depression on electrocardiogram (ECG), occurrence of sharp piercing pain over the center or left side of the chest, which was generally more intense at breathing, shortness of breath when reclining, presence of heart palpitations, low-grade fever, an overall sense of weakness, fatigue or feeling sick, cough, and abdominal or leg swelling. For both the patients and controls, we used a nominal scale of positive (zero to five), being zero as no symptoms or no clinical findings, and five as the strongest symptoms. Only a few patients were examined by chest X-ray for an enlarged cardiac silhouette indicating pericardial effusion.

### 2.3. Histopathology (H&E Stain), Direct Immunofluorescence (DIF), Indirect Immunofluorescence (IIF), Confocal Microscopy (CM) and Immunohistochemical (IHC) Studies

Pericardial tissue samples were obtained from seven El Bagre-EPF patients and the matched controls at necropsies in the local hospital.

Because our previous studies showed that the sera of El Bagre-EPF patients also bind to the intracellular, intranuclear, cytosolic, and membrane-attached proteins, we prefixed the section of cow tissues on the slides with freshly prepared 3.5% paraformaldehyde, washed with phosphate-buffered saline (PBS), permeabilized the tissue by treating the slides with 0.1% Triton X-100 and 5% goat serum in PBS for 10 min. After further washing, the tissue sections were incubated with the patient’s serum for one hour, washed with PBS again, and incubated with the secondary antibody for 1.5 h. For detailed methods see Supplementary Materials. Detailed methods for histopathology (H&E stain), DIF, IIF, CM, and IHC are also described in Supplementary Materials. Table 1 shows the catalog numbers and used dilutions for the antibodies in the IHC study, with IHC stain strength (highest +++ to lowest −), according to the method reported by McCarty et al. [17].

### 2.4. Statistical Analysis

We used the Fisher exact test to compare two nominal variables (e.g., positive and negative) of antibody response. *p* < 0.05 with a 95% confidence or more was considered statistically significant. We used the software GraphPad QuickCalcs (version 9.1, GraphPad Software Inc., La Jolla, CA, USA). Additionally, we used autoantibodies in the diagnosis of El Bagre-EPF by assessing using the Chi-square test, and it was found to be statistically significant with a *p* value ≤ 0.05 (Table 2 and Table 3).

## 3. Results

### 3.1. Clinical Symptoms of the El Bagre-EPF Patients

Although the differences in clinical symptoms between the patients and controls were subtle, as demonstrated in Table 2, the patients of El Bagre-EPF predominantly showed episodes of sudden chest pain, dyspnea, hiccups, dysphagia, palpitations, fatigue, anxiety, confusion, hoarseness, and syncope without respiratory infections. One patient had active tuberculosis, which was treated. Active tuberculosis was not seen in the controls. The most common clinical symptom was sharp piercing chest pain over the center of the chest, especially painful when breathing. This was observed in 33/45 patients, and, using the Chi-square test, was found to be statistically significant with a *p* value ≤ 0.05.

### 3.2. Results of Immunohistochemistry for Patient Tissues (IHC)

IHC studies showed heterogeneous and polyclonal reactivities in tissue samples from El Bagre-EPF patients and labeled epi-pericardial tissues, both blood and lymphatic vessels, nerves, and pericardial sensory nerve formations. No positive immunoreactivity was detected in disease-free control cadaveric necropsies (Table 1). Strong immunoreactivity in IHC studies was observed by using specific antibodies against human fibrinogen (7/7), kappa chain (7/7), lambda chain (7/7), human albumin (7/7), human IgG (6/7), IgM (6/7), human complement/C3c (5/7), human complement/C3d (5/7), C5b-9 complement (5/7), human IgD (4/7), human C1q (4/7), human IgA (0/7), and human IgE (0/7) in El Bagre-EPF patients. (*p* < 0.1).

Of interest, most of the patients presented with autoantibodies in a polyclonal way to both the cell junctions as well as those to the neural receptors. This was observed in 42/45 patients, and, using the Chi-square test, was found to be statistically significant with a *p* value ≤ 0.05.

### 3.3. Results of Immunofluorescence Tests (DIF and IIF) and Confocal Microscopy (CM)

The results of IIF and CM on cow’s epi-pericardial as antigen source are shown in Supplementary Materials. In both IIF and CM microscopy studies on El Bagre-EPF patients, positive reactivities with epi-pericardial tissues were seen by utilizing antibodies against human fibrinogen (43/45), kappa chain (45/45), lambda chain (45/45), human albumin (42/45), human IgG (43/45), IgM (42/45), human complement C3c (38/45), IgD (25/45), C1q (35/45), IgA (0/45), and IgE (1/45) (*p* < 0.1). None of the control cadaveric necropsies showed positive reactivity.

**Figure 1:**
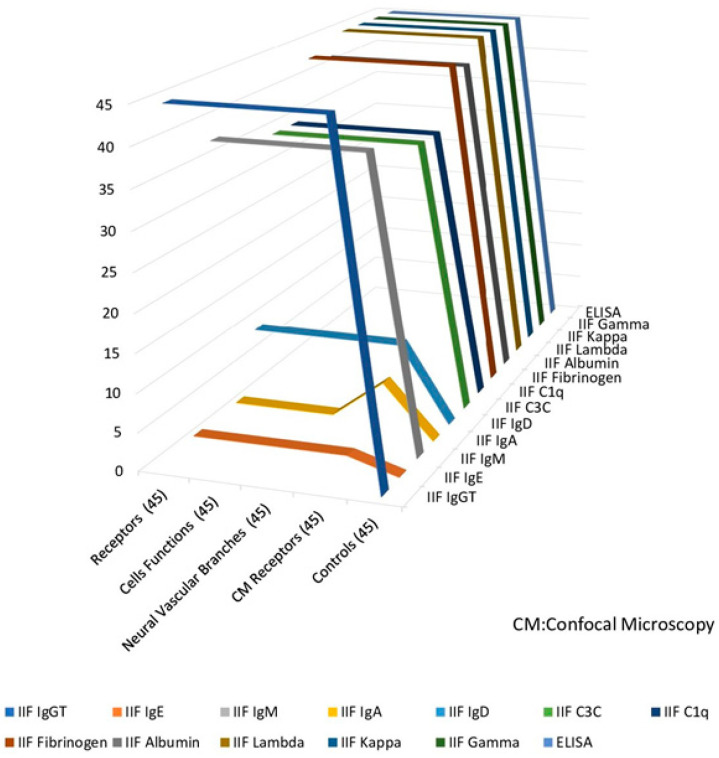
A graphic representation of the summarized results for the presence of antibodies to the epicardium, pericardium, and their respective sensory nerve formations as neurovascular structures. Our findings prove our hypothesis that the patients have autoantibodies to the cell junctions on the epi-pericardium in a polyclonal fashion.

In Figure 1, we present a summarized graphic representation of the results of the positive reactivity of each antibody with the epicardium, pericardium, the respective nerve profiles, and associated sensory nerve formations, as well as neurovascular bundles. These antibodies included those against total IgG (IgG T), IgM, IgD, kappa chain, gamma chain, C3c of the complement, C1q, fibrinogen, and albumin (*p* < 0.1). None of the patients were positive for the layers of the pericardium, the neurovascular branches, or the sensory nerve formations for these antibodies (*p* < 0.1).

**Figure 2:**
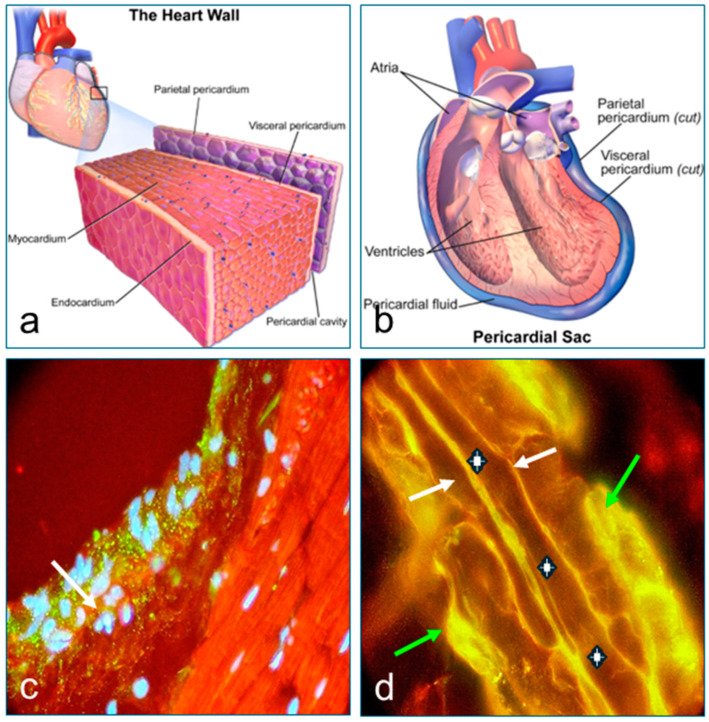
This is a (**a**) graphical representation of the pericardium. Source: Wikipedia. A representation of the pericardium, heart, and vessels. Credentials and courtesy to: (https://en.wikipedia.org/wiki/Pericardium#/media/File:Blausen_0724_PericardialSac.png). (**b**) A graphic representation showing in more detail the layers of the pericardium and their relationship with the heart muscle. Courtesy and credits to: (https://en.wikipedia.org/wiki/Pericardium#/media/File:Blausen_0470_HeartWall.png). (**c**) Positive staining to cell junctions of a cow heart, using FITC conjugated antibody (Ab) to IgG and Ab to a MYZAP. The positive stain to multiple cell junction proteins (yellow points) in both layers of the pericardium was observed. The white cross indicates the separation of the pericardium from the myocardium for the solubilizing buffers (1000×). (**d**) Positive stain to both the external and internal pericardium membranes, using the FITC conjugated Ab to IgG that colocalized with the reactivity of Ab to an ARVCF (yellowish stain, white arrows) (1000×). The positive stain to multiple cell junctions (yellow points) in both layers of the pericardium was seen. The white cross shows the virtual space between the pericardial layer’s buffers. With these studies, we were able to demonstrate the presence of autoantibodies to several structures in the epicardium proving our hypothesis.

**Figure 3:**
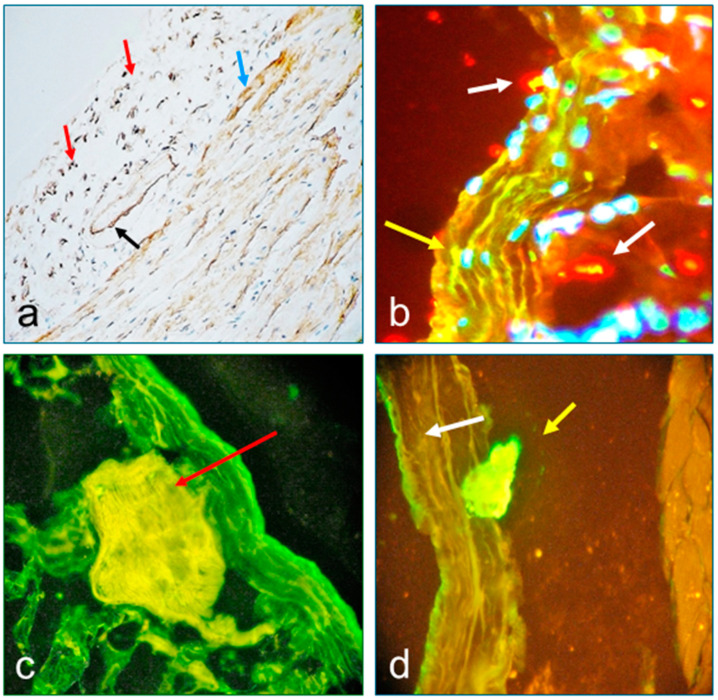
(**a**) Immunohistochemical (IHC) stain using the antibody to C3c on a patient necropsy showing positive stain in the epicardium cells junctions (dark points, red arrows). A positive stain was also seen on the envelope of a large vessel inside the epicardium (brown stain black arrow) and on the adjacent heart muscle (light brown stain, blue arrow) (200×). (**b**) Confocal microscopy showed a positive colocalized stain with FITC conjugated antibody (Ab) directed to IgG and with the Ab to MYZAP (400×). The white arrows indicate positive reactivity against several components of the sensory nerve formations. The yellowish stain (yellow arrows) is the positivity to the epicardium and pericardium. The nuclei are stained with DAPI. (**c**) Positive stain to a large neurovascular bundle under the pericardium (yellow stain) (red arrow) using the FITC conjugated Ab to fibrinogen (400×). (**d**) A confocal image demonstrated positive staining to sensory nerve formations by using the FITC conjugated Ab to IgG, which colocalized with the staining of ARVCF (yellowish stain, yellow arrow). The white arrow indicates the positivity between the epicardium and the pericardium. The structure on the right is the myocardium (1000×). The sera from most El Bagre-EPF patients displayed polyclonal autoreactivity, including cell junctions and sensory nerve formations, demonstrating our hypothesis.

**Figure 4:**
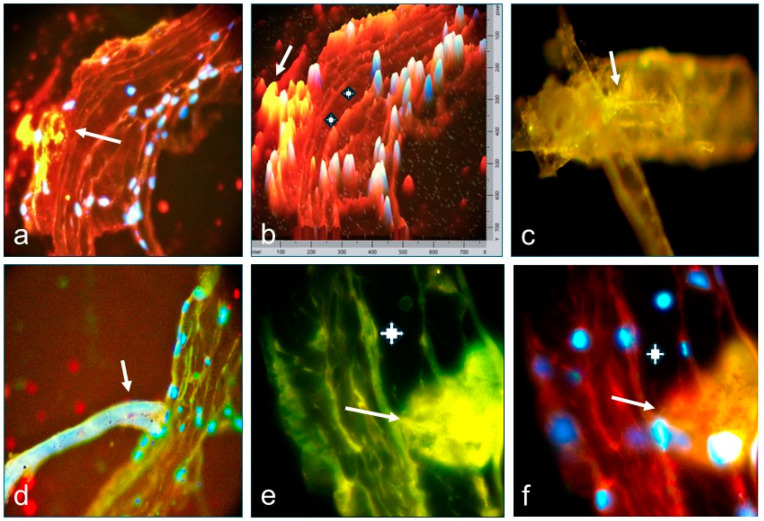
(**a**–**e**) Confocal images (all at 1000×). (**a**) Image shows a positive stain to a membrane receptor in the outside layer of the pericardium using the FITC-conjugated antibody (Ab) to fibrinogen, which was colocalized with the staining of Ab to ARVCF (yellowish stain, white arrow). (**b**) A two-dimensional confocal image of (**a**) that clearly showed the anatomical area of the membrane receptor (yellowish stain) (white arrow), and the two white crosses show the virtual space between the two layers of the pericardium. The blue is DAPI. (**c**) Positive stain to a vessel attached to the external layer of the pericardium using the FITC-conjugated Ab to IgM, which was colocalized with the staining of Ab to desmoplakin I II, with details of membrane and intracellular elements (white arrow, yellowish stain). (**d**) The white arrow shows a positive stain on another vessel attached to the external pericardium using the FITC-conjugated Ab to IgM, which was colocalized with the staining of Ab to p0071. (**e**,**f**) Staining to other morphotypes of sensory nerve formations (white arrows), being projected towards the virtual space between the two layers of the pericardium. The antibodies from other patients were used. The white cross indicates the virtual space among the pericardium layers. We counterstained the nuclei with DAPI. This membrane receptor seems to have two nuclei, or one is near the attachment to the pericardium. Our findings prove our hypothesis that the patients have autoantibodies to the cell junctions on the epi-pericardium in polyclonal fashion and that the autoantibodies perfectly colocalize with the commercial antibodies.

In Figure 2, Figure 3 and Figure 4, we present the results of the commercial antibodies directed to MIZAP, ARVCF, desmoplakin I-II, and p0071, which were perfectly colocalized those with the autoantibodies to El Bagre-EPF autoantibodies, i.e., the membrane receptors were positive in the epicardium, the pericardium, the neurovascular bundles feeding, and their respective cells junctions (*p* < 0.1).

## 4. Discussion

The present study was designed to analyze whether the pericardium of El Bagre-EPF patients is a target for the autoantibodies of these patients since they usually present thoracic symptoms. Although the clinical manifestations of the disease El Bagre-EPF are primarily cutaneous lesions, it has been shown that it can also affect other organs, including the cardiovascular system [10,11,12,13]. In the present study, we found that El Bagre-EPF autoantibodies bind to the pericardium (both fibrous and serous), the pericardial vessels and nerves, and structures resembling sensory nerve formations reported in the pericardium of dogs [18,19], which are mechanoreceptors or chemoreceptors [20,21]. Our patient autoantibodies colocalized 100% with the illegally commercialized Progen antibodies, as documented above in methods.

The pericardium is a double-walled membrane (fibrous and serous) that envelops the heart and the origin of the great blood vessels. The fibrous pericardium is a thin membrane that consists of connective tissue cells, large collagen bundles, and elastic fibers and contains abundant vessels and nerves [22,23,24]. On the other side, the serous pericardium is formed by two layers. The inner membrane or epicardium covers the surface of the myocardium and consists of a layer of flattened mesothelial cells [25], while the outer membrane covers the inner surface of the fibrous pericardium, which is formed by a plane layer of mesothelial cells. Both layers of the serous pericardium limit a virtual pericardial space filled with liquid [25].

To understand the involvement of the pericardium in El Bagre-EPF, it is necessary to explain its structure. The mesothelial pericardial cells have microvilli and scarce cilia in the luminal surface [25] and show numerous junctional cell membrane complexes in the skin (i.e., gap junctions, desmosomes, adherens, and tight junctions) whose structural proteins are targets for El Bagre-EPF autoantibodies [10,11,12,13,16,26]. The results of the present study for the pericardium strongly support that junction cell proteins are targets for El Bagre-EPF autoantibodies independently of their localization in the pericardium. Mesothelial cells in the peritoneum can produce and secrete mixed molecules in response to exterior signals, that regulate inflammatory responses, recruit immune cells into serosal cavities, and present antigens to specialized T cells [27]. They also play a role in removing fibrin from the serosal cavities [26]. Both fibrinogen and albumin are regarded as protectors of tissue damage, independent of the nature of the tissular lesion origin or alteration on those compounds [28]; in addition to cell-junction membrane proteins, further affinity chromatography studies using albumin and or fibrinogen and testing the patient’s sera will be required to study their role in El Bagre-EPF pathogenesis. Due to the lack of examinations by specialized MRI and echocardiogram, only the clinical symptoms, signs, EKG, and chest X-ray for a few cases were examined, which showed a slight positivity to pericardial disease in the patients (Table 3). The small hospital in the endemic area often has an unworking X-ray machine and does not have an echocardiogram or magnetic resonance imager (MRI). In addition, health insurance does not approve of sending patients to cities with those resources.

In general, pericardial heart disease is observed more frequently at necropsy than in live patients [29,30]. Indeed, older literature mentioned that pericarditis is an inflammation of the pericardium. Dr. Pierre Louis Alphée Cazenave, who described pemphigus foliaceus for the first time [31], reported in his seminal book that multiple patients who died before or around the time when corticosteroids were just beginning to be used for pemphigus and pemphigoids. In the same book, in many patients with pemphigus, necropsies presented with serositis, peritonitis, and pericarditis, and also showed the presence of granulation tissue, resembling chronic inflammation, around most of the serous layers in several organs [31]. He also detected multiple old adherences in several organs covered by serous membranes and diaphragm [31]. Similar findings were described by others [32]. Indeed, before the use of corticosteroids for autoimmune skin blistering diseases, serosites, such as pericarditis, pleuritis, and peritonitis, were described in patients with pemphigus vulgaris and dermatitis herpetiformis. Again, most of these findings were shown in post-mortem [31,32,33,34]. Our findings suggest that autoreactivity to endocardial and pericardial components causes pericarditis, which was detected in a few patients with chronic uncontrolled disease, as well as in the necropsies.

Thus, based on our IHC findings, El Bagre-EPF patients may undergo clinical or subclinical pericarditis, as well as symptoms and signs, before and around the implementation of corticosteroids as treatment [31,32,33,34].

Our finding reinforces previous studies indicating that autoantibodies of the El Bagre-EPF patients recognize cell junction proteins present in the endoneurial, perineurial, and epineural sites of the nerves. In the present study, we also demonstrated that encapsulated formations in the sensory nerve formations have some type of small cell junctions recognized by the patient autoantibodies. Indeed, other authors have previously shown the presence of Ruffini-like corpuscles (RLCs) in all pericardia and allocated to three histologic sensory nerve formations depending on the fibrous pericardium layering in precise anatomical sites [35].

The use of paraformaldehyde in our studies as a crosslinker fixative helped to cross-link cell membranes and cytoplasmic proteins [36]. The simultaneous use of Triton X-100 (C_14_H_22_O(C_2_H_4_O)_n_), a nonionic surfactant (detergent), is useful to solubilize and separate biomembrane components, especially to separate underlying detergent–lipid interactions and “open pores” on the cells membranes, that are bilayers composed mostly of lipids [36]. We noticed that this combination of paraformaldehyde, Triton X-100, and blocking agents, such as goat serum (all very timely, and temperature controlled), is excellent for exposing epitopes on unknown lipid-proteins, which may be glycosylated and phosphorylated and may have other possible conformational posttranscriptional modifications. Detergents interact with biomembranes (cell junction molecules in our study) due to their amphiphilic character to intercalate between lipids [37].

Nevertheless, caution is needed in drawing conclusions from our findings, as they are based solely on clinical findings (and not always complete), and the results of immunofluorescence tests and IHC. One main limitation of our study is the absence of various clinical tests, particularly radiological tests. These tests, except with the chest X-ray (when the machine is working), are not available in the very rural area of El Bagre. Furthermore, the Colombian Health Promoting Entity healthcare system has been broken for years and rarely accepts admissions to hospitals for people in severe poverty.

On the other hand, fibrinogen deposits are a hallmark of tissue damage, despite the triggering nature of such wounds, insults, or infections (by any microorganism and or virus), as well as by immunological causes [28]. Fibrin can curb the properties of an inflammatory response, including attracting leukocytes from the bloodstream [28,38,39]. Therefore, we may hypothesize that the presence of albumin and fibrinogen may play roles as transporters and initial blockers, which try to protect the damaged structures.

## 5. Conclusions

For the first time in the world literature, we found that ARVCF, MYZAP, desmoplakin, and p0071 are present in the complex perineurium, its membrane, and sensory nerve formations, which are also reacted by the patients’ autoantibodies. Our use of the partial permeabilization of membranes and the use of detergents allowed us to discover these findings. Pericarditis is usually a postmortem diagnosis. At this point, we do not have answers to the functional and/or pathological effects of the autoantibodies on the membrane and chemoreceptors in the pericardium and to any clinical symptoms in the patients. Our study greatly improves the understanding of the field of the pleura and provides a new set of antibodies to study the pericardium and its sensory nerve formations. Material Transfer Agreements should always be signed between researchers, independent of the “trust” between the parties when handling biological materials.

## Figures and Tables

**Table 1:** The results of the immunohistochemical (IHC) stain studies for the epicardium samples from the cadaveric patients and controls with the antibodies and their catalog numbers. E/P: Positivity epi/pericardia; SNF: sensory nerve formations; P: summary from patients; C: summary from controls. Readings were recorded as negative (−), or positive (++, or ++++) depending on the intensity of the staining reactions. All tests were repeated and titrated at least twice. The titers in replicate tests sometimes varied by +1 dilution. The results reported are the range of values obtained or median titers. The interpretation of readings adopted was the following (writing a ‘+’ for a reading of −, ++, +++ or ++++), being ++++ the highest stain.

Catalogue Number	IHC All the Products from Agilent-Dako	E/P^1^ P	SNF^2^ P	E/P C	SNF Cs
AR0423	Polyclonal rabbit anti-human IgG (Range 1:250 to 1:500)	(+++)	(+++)	(−)	(−)
IR513	Polyclonal rabbit anti-human IgM (Range 1:150 to 1:300). Flex ready to use.	(+++)	(+++)	(−)	(−)
IR510	Polyclonal rabbit anti-human IgA (Range 1:100 to 1:200). Flex ready to use. High Ph antigen retrieval.	(−)	(−)	(−)	(−)
IR517	Polyclonal rabbit anti-human IgD Flex ready to use.	(++)	(++)	(−)	(−)
A0094	Polyclonal rabbit anti-human IgE. (Range 1:750 to 1:1500).	(−)	(−)	(−)	(−)
A 0062	Polyclonal Rabbit anti-human C3C complement (Range 1:100 to 1:200).	(+++)	(+++)	(−)	(−)
M077	Monoclonal mouse anti-human C5b-9 complement (Range 1:25 to 1:50). (MAC).	(+++)	(+++)	(−)	(−)
A0063	Polyclonal rabbit anti-human C3d- complement (Range 1:400 to 1:800).	(+++)	(+++)	(−)	(−)
A0136	Polyclonal rabbit C1-q Complement (range not provided).	(++)	(++)	(−)	(−)
A0001	Polyclonal rabbit anti-human Albumin. (Range 1:2000 to 1:4000).	(++++)	(++++)	(−)	(−)
A0080	Polyclonal rabbit anti-human fibrinogen (Range 1:200 to 1:400).	(++++)	(++++)	(−)	(−)
IR507	Polyclonal rabbit anti-human Lambda. Flex ready to use. Heat induced epitope retrieval	(++++)	(++++)	(−)	(−)
IR506	Polyclonal rabbit anti-human Kappa light chains Flex ready to use. Heat induced epitope retrieval	(++++)	(++++)	(−)	(−)
GA60066-2	Negative control. Rabbit, FLEX RTU. Control reagent, Ready-to-use, Unconjugated for Immunohistochemistry,	(−)	(−)	(−)	(−)

**Table 2:** The results of clinical and radiological tests for symptoms and/or signs associated with a possible diagnosis of pericarditis. Nominal scale used zero (negative) to five (strongest).

Symptoms/Signs, EKG/Chest RX	Patients	Controls
Sharp piercing chest pain over the center or left side of the chest, which is generally more intense when breathing.	33/45	30/45
Continuous dyspnea.	7/45	0/45
Pericardial rub on auscultation.	2/45	0/45
Hiccups.	7/45	2/45
Dysphagia.	10/45	3/45
Hoarseness and syncope without respiratory infections.	7/45	2/45
Current smokers.	2/45	345
Past smokers.	8/45	9/45
Palpitations.	38/45	12/45
Fatigue.	45/45	16/45
Weakness.	39/45	1/45
Shortness of breath when reclining.	7/45	0/45
Previous history of viral infections previous the above symptoms.	45/45	45/45
Abdominal or leg swelling.	7/45	1/45
Cardiomegaly using chest X Ray.	19/45	3/45
New widespread ST-elevation or PR depression on ECG	12/45	1/45
Pleural infusion by X-ray	0/45	0/45

**Table 3:** Results of indirect immunofluorescence (IIF) and confocal microscopy (CM).

TEST Antigen Source Cow	P-Ab Sensory Nerve Formations at E/P (45)	P-Ab Functions at E/P (45)	Patients-Ab to Neural Epidermal Branches at E/P (45)	CM P Sensory Nerve Formations at EP (45)	Controls (45)
IIF IgG total	43	43	43	43	0
IIF IgE	1	1	1	1	0
IIF IgM	42	42	42	42	0
IIF IgA	0	0	0	0	0
IIF IgD	25	25	25	25	0
IIF C3C	38	38	38	38	0
IIF C1q	35	35	35	35	0
IIF Fibrinogen	45	45	45	45	0
IIF Albumin	42	42	42	42	0
IIF Lambda	45	45	45	45	0
IIF Kappa	45	45	45	45	0
Confocal IIF IgG total	43	43	43	43	0
Confocal IIF IgE	1	1	1	1	0
Confocal IIF IgM	42	42	42	42	0
Confocal IIF IgA	0	0	0	0	0
Confocal IIF IgD	25	25	25	25	0
Confocal IIF C3C	38	38	38	38	0
Confocal IIF C1q	35	35	35	35	0
Confocal IIF Fibrinogen	45	45	45	45	0
Confocal IIF Albumin	42	42	42	42	0
Confocal IIF lambda	45	45	45	45	0
Confocal IIF Kappa	45	45	45	45	0

## Data Availability

All the data is available inside this publication.

## Supplementary Materials

The following supporting information can be downloaded at: https://www.mdpi.com/article/10.3390/diagnostics15080964/s1. Refs [40,41,42] are cited in Supplementary Materials file.

## Abbreviations

The following abbreviations are used in this manuscript:

EPF	Endemic pemphigus foliaceus
El Bagre-EPF	Endemic pemphigus foliaceus in El Bagre
PF	Pemphigus foliaceus
FS	Fogo selvagem
H&E	Hematoxylin and eosin
DIF, IIF	Direct and indirect immunofluorescence
BMZ	Base membrane zone
ICS	Intercellular stain between keratinocytes
PAF	Paraformaldehyde
FITC	Fluorescein isothiocyanate
DAPI	4′,6-diamidino-2-phenylindole
Dsg1	Desmoglein 1
CFM	Confocal microscopy
P0071	Plakophilin 4
ARVCF	Armadillo repeat gene deleted in Velo-Cardio-Facial syndrome
DP I-II	Desmoplakins I and II
MYZAP	Myocardium-enriched zonula occludens-1-associated protein
Abs	Antibodies
ARVCF	Arrhythmogenic right ventricular cardiomyopathy
ECG	Electrocardiogram
