# Supplementary material for: The Pericardium Cells Junctions Are a Target for Autoantibodies of Patients Affected by a Variant of Endemic Pemphigus Foliaceus in El Bagre and Surrounding Municipalities in Colombia, South America
Source: Diagnostics (Basel). 2025 Apr 10;15(8):964. doi: 10.3390/diagnostics15080964 (PMC12025448; doi:10.3390/diagnostics15080964)
Supplement: Supplementary file 1 [file diagnostics-15-00964-s001.zip › diagnostics-3517402-supplementary.pdf]

Supplementary Materials: Materials and Methods

Tissues used: Human cadavers from the autopsy material did not require Institutional Review Board (IRB) in El Bagre endemic area since cadavers and their specimens does not meet the regulatory definition of 'human subject research,' i.e., "a living individual and or a person" but an approval of the Relatives of the patients regarding -Privacy- was obtained including no identifiers.

A physical examination, and a questionnaire were filled out searching for the symptoms of episodes of sudden chest pain, pericardial rub on auscultation, dyspnea, hiccups, dysphagia, palpitations, fatigue, anxiety, confusion hoarseness and syncope.[1] During clinical examination, the following signs and symptoms were considered: stethoscope testing for a pericardial friction rub, previous history of viral infections, electrocardiogram (ECG) evidence of PR depression or ST segment deviation, occurrence of sharp, piercing chest pain over the center or left side of the chest, which is generally more intense when breathing in; shortness of breath when reclining, presence of not of heart palpitations; low-grade fever, an overall sense of weakness, fatigue or feeling sick, cough, abdominal or leg swelling. New widespread ST-elevation or PR depression on ECG We use a nominal scale of positive (zero to five), being zero, no symptoms or clinical findings, and five the strongest in both the cases and controls.

Laboratory testing: We tested 45 sera from patients with El Bagre EPF, and 45 normal control sera from the endemic area where the disease prevails. The patients were matched to controls by demographic aspects including age, gender, diet, and work activities. An interview, and a complete clinical history and physical exam were performed for all the subjects of the study. A human quality assurance review board approved the studies at the Hospital Nuestra Señora del Carmen in El Bagre. The participants signed informed consent forms, and the patients were evaluated clinically, by skin biopsy H&E staining as well as by direct and indirect immunofluorescence (DIF, IIF). Additional confocal microscopy (CFM) studies were performed as described[10,11,13,17,41] Only patients who satisfied the full diagnostic criteria for El Bagre-EPF were included, as follows: (i) patients exhibited clinical and epidemiological features described for this disease; (ii) patients lived in the endemic area; (iii) the patient autoantibodies displayed intercellular staining between epidermal keratinocytes using antihuman IgG, IgM, Kappa, Lambda, IgG4 as well as Complement/C3 as well as fibrinogen and albumin using DIF as well as IIF. Additional stains could be seen such as epidermal pericytoplasmic and/or dotted stains between the keratinocytes using anti-human IgD, fibrinogen and albumin antibodies. Positive staining with the antibodies also included a dotted stain between the basal cell junction at the basement membrane zone (BMZ) of the skin by either DIF or IIF as previously reported[2-7] Also, the presence of positive strong dotted staining could be seen in the lower part of the BMZ with the above antibodies. An additional polyclonal immune response could be seen in the corneal layer, as dotted ICS, at the BMZ or against skin dermal appendices, mainly directed to the cell's junctions and their neurovascular supply routes. Positive staining could also be seen at mesenchymal -endothelial cells junctions. Additional diagnostic criteria of El Bagre-EPF disease include (iv) the patient serum was positive by immunoblotting (IB) assay reactivity against desmoglein (Dsg)1, as well as for envoplakin, periplakin and BP230 plakin molecules as previously described[11,13][4-6];(v) the patient serum immunoprecipitated a Concanavalin A affinity-purified antigen bovine tryptic 45-kDa ectodomain fragment of Dsg1[11,13];and (vi) the patient serum yielded a positive result using an ELISA when screening for autoantibodies to El Bagre-EPF antigens[4-6] Colocalization of the autoantibodies could be seen with desmoplakin I-II (DP-I–II), plakophilin 4 (p0071), Armadillo Repeat gene deleted in Velo-Cardio-Facial syndrome (ARVCF), and myocardium-enriched zonula occludens-1-associated protein (MIZAP).

Hematoxylin and eosin stain (H&E) and immunohistochemistry (IHC) stains . We performed H&E and IHC stains as previously described[10,11,13,41].All samples were run with positive and

negative controls. The interpretation of IHC was based on overall staining intensity15: intensity (0: no immunoexpression; 1+: weak immunoexpression; 2+: moderate immunoexpression; and 3+: strong immunoexpression first developed by McCarty et al and termed an H&E score and others [17,42]. For our IHC staining we utilized a Leica (Buffalo Grove, IL) staining system. Specifically, for primary staining we utilized a Bond Max platform autostainer with bond polymer refined detection DS9800, a horseradish peroxidase linker polymer, and DAB chromogen (brown staining). Positive and negative controls were consistently included for study. For IHC, we utilized antibodies for complement/C5b-9 at the dilution of 1:50, without antigen retrieval (Dako; Agilent Technologies, Santa Clara, CA).

Direct immunofluorescence (DIF) on necropsies: DIF: In brief, we incubated a 4 μm thickness frozen skin section using PBS with 0.1% Triton X-100 and 1% normal goat serum for five minutes for partial permeabilization, to detect cytoplasmic, nuclear, and membrane binding putative antigens, and for blocking non-specific staining. The slides were then washed with PBS[10,11,13,41].The nuclei of the cells were counterstained with 4,6-diamidino-2-phenylindole (DAPI, Pierce; Rockford, IL, USA). We used antibodies to ARVCF, source guinea pig, Cat. no. GP155; Progen Biotechnik (Heidelberg, Germany). For its secondary, we used Alexa Fluor®555 goat-anti-guinea pig (Molecular Probes Life Technologies/ThermoFisher Scientific; Waltham, MA, USA). All samples were run with positive and negative controls. We classified our findings as negative (-), weakly positive (+), moderately positive (++) and strongly positive (+++)

Indirect immunofluorescence (IIF): Our studies were performed as previously described[10,11,13,17,41] Human and bovine tissue including epi/pericardial tissue cut at different areas. Mature cows were obtained from the abattoir within 2 hours of slaughter. Tangential sections were cut consecutively, in the plane tangential areas. Serial sections were taken at intervals of 2 slices (4 μm thick) as antigen sources for the IIF. These were incubated for 4 minutes (min) with 1X PBS and 3.5% paraformaldehyde (partial fixation). The slides were then washed twice with PBS for 10 minutes per wash, then partially permeabilized using 1X PBS with 0.1% Triton X-100 and 1% normal goat serum (for blocking and permeabilization) for 10 min under rotation and rewashed again twice with PBS. Slides were counterstained with 4′,6-diamidino-2-phenylindole (DAPI) (Pierce, Rockford, IL, USA). For colocalization, we used commercial antibodies directed to DP-I–II (mouse monoclonal multi-epitope cocktail, Progen Cat. no. 65146) dilution 1:50, ARVCF polyclonal antibody, (source guinea pig, tested in human and bovine) catalog no.GP155 (dilution 1:50), and for its secondary, we used Alexa Fluor®555 goat-anti-guinea pig from Molecular Probes/Life Technologies/Thermo Fisher Scientific (Waltham, Massachusetts, USA). We also used plakophilin-4 (dil 1:50), Cat. no.651166, and a mouse monoclonal antibody for Myozap; Cat no.651169. As a secondary antibody for DP-I–II, the p0071 and the Myozap, we used Texas red-conjugated goat anti-mouse IgG from Thermo Fisher. The antibodies to DP-I-II, ARVCF, p0071, and MIZAP were all obtained from Progen Biotechnik in Germany. For gap cell junction colocalizations, we used Connexin 43 (C43) produced in rabbit (**C6219), at 1:50** dilution (Sigma Aldrich, Saint Louis, Missouri, USA). As its secondary we used Rhodamine-conjugated affinity purified goat & rabbit IgG (FC) at 1:100 dil. from Rockland laboratory Inc., (Cat. no 611-1003). The samples were consistently run with positive and negative controls. We classified our findings as negative (−), weakly positive (+/−), positive (++) and strongly positive (+++). Two separate investigators with inter-rater reliability assessments evaluated the experiments.

Confocal microscopy studies (CFM): Performed as previously described [11,13,17]. Standard 20X, 40X and 100X objective lenses were used. Each frame included an area 440 to 330 mm. Images were obtained using EZ-1 image analysis software (Nikon, Tokyo, Japan). For colocalization experiments with the serum autoantibodies, we used the antibodies to DPI-II, ARVCF, p0071, and MYZAP.

Immunohistochemistry (IHC): To colocalize patient autoantibodies, we also utilized IHC with a dual endogenous peroxidase blockage and an Envision dual link, according to Dako specifications.

We tested for anti-human IgG, IgA, IgM, IgD, IgE, Complement/C1q, Complement/C3c, Complement/C3d, albumin, fibrinogen, kappa, lambda, (all from Agilent Dako).

Statistical analysis: We used Fisher's exact test to compare two nominal variables (e.g., positive and negative) of antibody response. We also compared the differences when evaluating: (i) positivity of the El Bagre-EPF autoantibodies between patient cases and controls; and (ii) patient antibody results versus the commercial antibodies to Myozap, p0071, DP-I–II and ARVCF. A $p < 0.05$ with a 95% of confidence or more was considered statistically significant. We used the software GraphPad QuickCalcs from GraphPad Software (La Jolla, California, USA).
